# The methyl-CpG-binding domain (MBD) is crucial for MeCP2’s dysfunction-induced defects in adult newborn neurons

**DOI:** 10.3389/fncel.2015.00158

**Published:** 2015-04-24

**Authors:** Na Zhao, Dongliang Ma, Wan Ying Leong, Ju Han, Antonius VanDongen, Teng Chen, Eyleen L. K. Goh

**Affiliations:** ^1^Programme in Neuroscience and Behavioral Disorder, Duke-NUS Graduate Medical SchoolSingapore, Singapore; ^2^Key Laboratory of Health Ministry for Forensic Science, Department of Forensic Medicine, Xi’an Jiaotong University School of MedicineXi’an, Shaanxi, China; ^3^Department of Physiology, Yong Loo Lin School of Medicine, National University of SingaporeSingapore, Singapore; ^4^KK Research Center, KK Women’s and Children’s HospitalSingapore, Singapore

**Keywords:** Rett syndrome, newborn neurons, dendrites, methyl-CpG-binding domain, spontaneous Ca^2+^ oscillations

## Abstract

Mutations in the human X-linked gene *MECP2* are responsible for most Rett syndrome (RTT) cases, predominantly within its methyl-CpG-binding domain (MBD). To examine the role of MBD in the pathogenesis of RTT, we generated two MeCP2 mutant constructs, one with a deletion of MBD (MeCP2-ΔMBD), another mimicking a mutation of threonine 158 within the MBD (MeCP2-T158M) found in RTT patients. MeCP2 knockdown resulted in a decrease in total dendrite length, branching, synapse number, as well as altered spontaneous Ca^2+^ oscillations *in vitro*, which could be reversed by expression of full length human MeCP2 (hMeCP2-FL). However, the expression of hMeCP2-ΔMBD in MeCP2-silenced neurons did not rescue the changes in neuronal morphology and spontaneous Ca^2+^ oscillations, while expression of hMeCP2-T158M in these neurons could only rescue the decrease in dendrite length and branch number. *In vivo* over expression of hMeCP2-FL but not hMeCP2-ΔMBD in adult newborn neurons of the dentate gyrus also rescued the cell autonomous effect caused by MeCP2 deficiency in dendrites length and branching. Our results demonstrate that an intact and functional MBD is crucial for MeCP2 functions in cultured hippocampal neurons and adult newborn neurons.

## Introduction

Rett syndrome (RTT) is a X-linked neurological disorder affecting mostly females. Classical RTT is a progressive neurodevelopment disorder. Girls with RTT exhibit normal development through the first 6–18 month after birth, followed by an abrupt neuroregression and growth stagnation (Neul et al., 2010). Mutations within *MECP2* (methyl-CpG binding protein 2) on the X-chromosome are responsible for nearly 95% of all RTT cases (Amir et al., 1999). MeCP2 is a member of the family of methyl-CpG binding domain (MBD) containing proteins that is the most abundant in post-mitotic neurons and it functions as a transcriptional regulator in the brain. MeCP2 contains a N-terminal domain (NTD), a methyl-binding domain (MBD), an intervening domain (ID), a transcriptional-repressor domain (TRD) and a C-terminal domain (CTD; Hansen et al., 2011). Common mutations found in RTT patients are primarily clustered within the MBD and the TRD of MeCP2 (Bienvenu and Chelly, 2006; Heckman et al., 2014). Patients with mutations in the MBD exhibit more severe clinical features than the mutations beyond this area (Fabio et al., 2014). The most common mutation found in RTT patients occurs at residue T158 located at the C-terminus of the MBD (Ballestar et al., 2005; Ghosh et al., 2010). Considering the key role of MBD in transcriptional function of MeCP2 and the high frequency of T158 mutations observed in RTT patients, the function of the MBD as well as the T158 mutation has become an important focus of many studies.

Abnormal levels of MeCP2 in the brains of mouse RTT disease models lead to RTT-like phenotypes including tremors, breathing abnormalities, hypoactivities and limb stereotypies. Like the human conditions, mice RTT models show an apparent normal early development before the onset of overt symptoms. After the onset of symptoms, the animals typically die at 10–12 weeks of age (Chen et al., 2001; Belichenko et al., 2008; Ricceri et al., 2013). Previous studies demonstrated that expression levels of MeCP2 in humans and rodents increase during neuronal development and maturation, suggesting MeCP2 may be important during the normal neuronal development and maturation (Shahbazian and Zoghbi, 2002; Nguyen et al., 2012; Ma et al., 2015). Studies in *Xenopus* also revealed a specific function of MeCP2 during early neural development (Stancheva et al., 2003; Marshak et al., 2012).

Brain autopsy material from RTT patients and MeCP2 mutant mice revealed normal gross anatomy without detectable loss of neurons but impaired dendritic growth and reduced complexity of pyramidal cells in the associate brain regions (Armstrong, 2005; Chapleau et al., 2009). This observation prompted the hypothesis that an underlying cause of RTT is a defect in neuronal and synaptic function. MeCP2 deficiency in cells and mice as well as cells from RTT patients are associated with changes in cellular and synaptic physiology (Marchetto et al., 2010; Ricciardi et al., 2011; Ma et al., 2015). However, it is still not clear if these cellular and synaptic changes and defects are cell-autonomous effects and if they are caused by the loss-of-function mutations in RTT neurons. Although it is possible to generate RTT mouse models with each individual human mutation identified, it will not be possible to distinguish between cell-autonomous and non-cell-autonomous (or secondary) effects of MeCP2 in neurons of these mice where MeCP2 is mutated or deleted in all cell types. Therefore, an *in vivo* system that allows genetic manipulation of individual cells in the brain is necessary to circumvent the limitations associated with all currently available MeCP2 knockout/knock-in mouse models of RTT.

Here we provide functional evidence on the MBD-dependent role of MeCP2 in neuronal development in cultured hippocampal neurons. Full length MeCP2 and mutant MeCP2 containing either the MBD deletion or T158→M mutation were used to study morphological and functional roles of the MBD in these neurons. Short hairpin RNA (shRNA) was targeted to individual newborn granule neurons in adult brain to determine cell-autonomous effects of MeCP2 *in vivo*.

## Materials and Methods

### Plasmid and Viral Production

For MeCP2 knockdown, a short hairpin RNA (GGGAAACTTGTTGTCAAGATGCC) was cloned under the control of the human U6 promoter with Tomato co-expressing under the Synapsin promoter. A shRNA with scrambled sequence was used as a control as described previously (Ma et al., 2015). In order to overexpress MeCP2 proteins in post-mitotic cells, a plasmid encoding the full length human wild-type *MECP2* (hMeCP2-FL) was generated under the control of the Ubiquitin promoter (Ub) in the lentiviral FUGW vector. Constructs expressing an MBD deletion (ΔMBD) or T158 mutation (T158M) were generated using the same lentiviral backbone. To examine the functional role of MeCP2 and its mutants in structural plasticity *in vivo*, engineered self-inactivating murine retroviruses were used to express GFP specifically in proliferating cells and their progeny in the dentate gyrus of adult mice. hMeCP2-FL or ΔMBD together with GFP were cloned under the control of EF1α promoter with shMeCP2 co-expressed under the control of human U6 promoter in the same vector. Western analysis was performed to validate the specificity and efficiency of the different constructs.

A high titer of virus (1 × 10^9^ unit/ml) was produced by transfection of different constructs into HEK293gp cells using the calcium phosphate method as described previously (Ma et al., 2015). Briefly, constructs were mixed with CaCl_2_ and added to 2 × HEPES buffer saline (pH = 7.0). The DNA mix was incubated for 30 min and then added to the HEK293gp cells followed by ultracentrifugation of viral supernatant.

### Primary Hippocampal Neuronal Culture and Transfection

Primary hippocampal neurons were isolated from embryonic day 18 (E18) Long-Evans rats and embryonic hippocampi were collected in buffer (127 mM NaCl, 5 mM KCl, 170 μM Na_2_HPO_4_, 205 μM KH_2_PO_4_, 5 mM Glucose, 59 mM Sucrose, 100 U/mL Penicillin/Streptomycin, pH 7.4). At least 3–4 batches of culture were used for each experiment, with at least 2 coverslips per batch. Each batch of cultures was isolated from pooled hippocampi of all E18 pups (typically 8–10) from 1 animal. Cells were dissociated with 25 mg/ml papain and plated on poly-L-lysine (1 mg/ml) coated coverslips or plates. High densities of cells (84000/well) were prepared for calcium imaging and low densities of cells (42000/well) were used for immunochemistry staining. Hippocampal neurons were cultured in Neurobasal medium (Invitrogen) supplemented with B-27, penicillin-streptomycin, L-glutamine at 37°C. Neuronal cultures were infected with the lentiviral vector carrying the control shRNA (shctrl) or MeCP2 shRNA (shMeCP2) construct at DIV (day *in vitro*) 1. Lentiviral vector carrying the rescue construct (hMeCP2-FL/ΔMBD/T158M) was added immediately after the red-fluorescent of shRNA was visualized (48 h after shMeCP2 transfection).

### Immunochemistry

After DIV 12, hippocampal primary neurons were fixed for 30 min with 4% paraformaldehyde in 0.1 M phosphate buffer (PB), washed with DPBS (Invitrogen) three times and then blocked with 5% normal donkey serum in 0.1% TBS-Triton (TBS-TX) buffer for 2 h at room temperature. Primary antibodies were diluted in blocking solution at 4°C using the following dilutions: 1:1000 rabbit anti-MAP2 (Millipore), 1:1000 mouse anti-MAP2 (Sigma), 1:1000 mouse anti-Synapsin-1 (Abcam), 1:500 rabbit anti-MeCP2 (Cell Signaling Technology) and 1:500 mouse anti-MeCP2 (Sigma). After incubation in primary antibodies overnight, coverslips were incubated in the appropriate secondary antibodies diluted in blocking solution for 2 h at room temperature.

The brain tissues were fixed 2 weeks after stereotaxic injection by vascular perfusion through the left ventricle of the heart with sequential delivery of 50 ml of saline and 60 ml of 4% paraformaldehyde in 0.1 M PB. Coronal brain sections (40 μm) were prepared and processed for immunostaining using the anti-DCX (Santa Cruz; 1:300) antibodies.

### Imaging and Neuronal Morphology Analysis

Both coverslips and brain slices were imaged on a Zeiss LSM 710 confocal system (Carl Zeiss) using a multi-track configuration. For *in vitro* studies, all values were obtained from at least 3 batches of culture of at least 2 coverslips per batch. At least 20 neurons from each coverslips were used for analysis. Quantification of the total dendritic length and total dendritic branch number as well as the number of synapsin-1 positive puncta of each cell were performed as previously described (Ng et al., 2013; Ma et al., 2015). Images were semi-automatically traced with NIH ImageJ using the NeuronJ plugin, generating data for the total dendritic length and total dendritic branch number. Quantification of synapsin-1 puncta was done manually on a pre-determined length of traced dendrite, and then presented on graphs as the number of puncta per μm dendrite. For *in vivo* studies, three-dimensional reconstructions of the dendritic processes on brain slices were made from Z-series stacks of confocal images. The projection images were traced with ImageJ using the NeuronJ plugin and total dendritic length and total dendritic number of each virus infected new born neuron (DCX+) in the granule cell layer were analyzed. Acquisition of all images as well as morphology quantification was performed under “blind” conditions. The distributions of the total dendritic length and branch number of each individual neuron under different conditions were shown in accumulative distribution plots or bar plots. Cumulative frequency is used to determine the number of observations (e.g., total neurite length of each neuron) that lie above (or below) a particular value in a data set. The cumulative frequency is calculated using a frequency distribution table. This method is defined as the percentage of observations falling in each class interval. Relative cumulative frequency (%) can be calculated by dividing the frequency of each interval by the total number of observations. An average of total 25–30 neurons from 4–6 animals (per experimental group) injected with virus carrying shRNA and/or expression construct were analyzed.

### Calcium Imaging and Peak Detection

After DIV 12, hippocampal primary neurons were washed twice with loading buffer (118 mM NaCl, 4.69 mM KCl, 4.2 mM NaHCO_3_, 1.18 mM KH_2_PO_4_, 0.8 mM MgCl_2_, 2.0 mM CaCl_2_, 20 mM HEPES, 30 mM glucose, pH = 7.4) and incubated with X-Rhod-1 (Molecular Probes/Invitrogen, Carlsbad, CA) with a final concentration of 1 μM for 30 min at 37°C. In order to remove excess dye, cells were washed with loading buffer twice and incubated for additional 20 min to equilibrate intracellular dye concentration and allow de-esterification of the dye. Time-lapse image sequences of 500 frames were acquired with a region of 512 × 512 pixels, with 488 nm (FITC) and 534 nm filters on a LSM 710 inverted fluorescence confocal microscopy (Carl Zeiss, Pte. Ltd., Singapore). Images were acquired with ZEN software (Carl Zeiss Pte. Ltd., Singapore). At least 20 GFP positive neurons were randomly selected for calcium imaging (as indicated in figure legends) from at least 2 coverslips/batch.

All the data analysis was done using MATLAB (Mathworks, Natick, MA). For each coverslip, more than 20 cells were selected to record the calcium intensity under fluorescence with a sampling rate of 1.56 s/frame. Peak detection was done in MATLAB according to previous studies (Marchetto et al., 2010; Ma et al., 2015). The amplitude of each peak is measured by the difference between the peak value and the baseline.

### Animals and Stereotaxic Injection

Ethics statement: All animal procedures and applicable regulations of animal welfare were in accordance with IACUC guidelines and approved by SingHealth IACUC, Singapore.

Adult (5–6 weeks old) female C57BL/6 mice were purchased from SingHealth Experimental Medicine Center (SEMC), Singapore, and kept in a temperature controlled environment (22 ± 2°C) with a 12-h light/dark cycle. Animals were deeply anesthetized and stereotaxically injected at four sites (0.5 μl per site at 0.25 μl/min) with the following coordinates (from bregma in mm): anterioposterior, −2; lateral, ±1.5; ventral, 2.2; and anterioposterior, −3; lateral, ±2.5; ventral, 3. A total of 60 animals were used and all efforts were made to reduce the number of animal used and also to minimize animal suffering.

### Statistical Analyses

All the data were expressed as mean ± SEM. Student’s *t*-test for two groups’ comparison and one-way ANOVA with a *post hoc* multiple comparison (Tukey test) were used to analyze the statistical significance between groups. The statistical significance was set at *P* = 0.05.

## Results

### Full-Length MeCP2 is Essential for the Maintenance of Normal Dendritic Development in Primary Hippocampal Neurons

**Figure 1:**
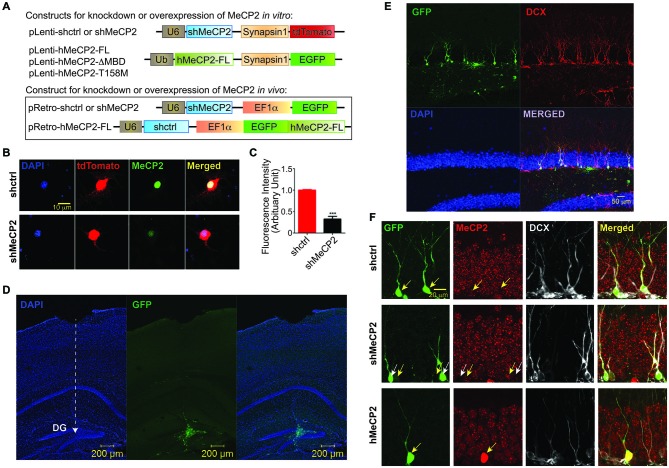
**Efficiency of MeCP2 knockdown in cultured primary hippocampal neurons. (A)** Schematic diagram showing lentiviral constructs for knocking down MeCP2 or expressing hMeCP2 (FL, ΔMBD or T158M) *in vitro* as well as retroviral constructs for checking the efficiency of MeCP2 knockdown or overexpression in progenitor cells *in vivo*. **(B)** Representative images from immunocytochemistry of MeCP2 in cultured hippocampal neurons at DIV 12 showing efficient knockdown of MeCP2 *in* *vitro*. **(C)** Quantification of fluorescence intensity of cells expressing shctrl or shMeCP2 that were immunostained with antibody against MeCP2 (***p < 0.001, Student’s *t*-test, *n* = 3 batches). **(D)** Low magnification (10x) images showing the virus injection site and needle track (dashed white line with arrow pointed at the end point of injection) in dentate gyrus (DG) of the mouse brain. **(E)** High magnification (20x) images showing the virus infected neurons co-localized with DCX (immature neuron marker). **(F)** Representative images showing efficiency of MeCP2 knockdown or overexpression in progenitor cells in the DG *in vivo* (Immature neurons identified with anti-doublecortin (DCX)). Virus-infected neurons (green) and their MeCP2 expression (red) were indicated on images with corresponding yellow arrows. shMeCP2-expressing cells in middle panel (pointed by white arrows) show no or very low expression of MeCP2 demonstrating efficient knockdown.

To investigate the role of endogenous MeCP2 in dendritic growth in cultured hippocampal neurons, we knocked down the expression of MeCP2 with lentivirus carrying shRNA against MeCP2 (Figure 1A). Endogenous MeCP2 protein level was significantly reduced in these neurons (Figures 1B,C). To confirm the efficiency of shMeCP2 *in vivo*, we generated retroviral construct carrying shMeCP2 (Figure 1A). An overexpression construct using the same retroviral backbone was also generated to express human MeCP2 (hMeCP2) that is not recognized by shMeCP2 specifically targeting mouse MeCP2 (Figure 1A). Retrovirus carrying either a knockdown construct or expression construct together with a fluorescence marker were stereotaxically injected into the border of hilus and DG where the progenitor cells are residing, as previously described (Ma et al., 2015) (Figures 1D–F). Retrovirus only infects dividing cells and therefore enables genetic manipulations (to express or knock down MeCP2) in individual progenitor cells in the adult hippocampus (Figures 1D–F). To confirm the neuronal identify of virus-infected cells, an immature neuronal marker, DCX was used for immunostaining (Figure 1E). The brain sections were also immunostained with antibody against MeCP2 to verify that virus-infected cells expressing fluorescence marker (GFP) express low or no MeCP2 (shMeCP2 expression construct) or express high MeCP2 (hMeCP2 expression construct) as compared to cells carrying shctrl expression construct (Figure 1F). This *in vivo* system also allows investigations on cell-autonomous effects of MeCP2 on neuronal development, which is not possible using any available knockout or knock-in RTT mouse models.

**Figure 2:**
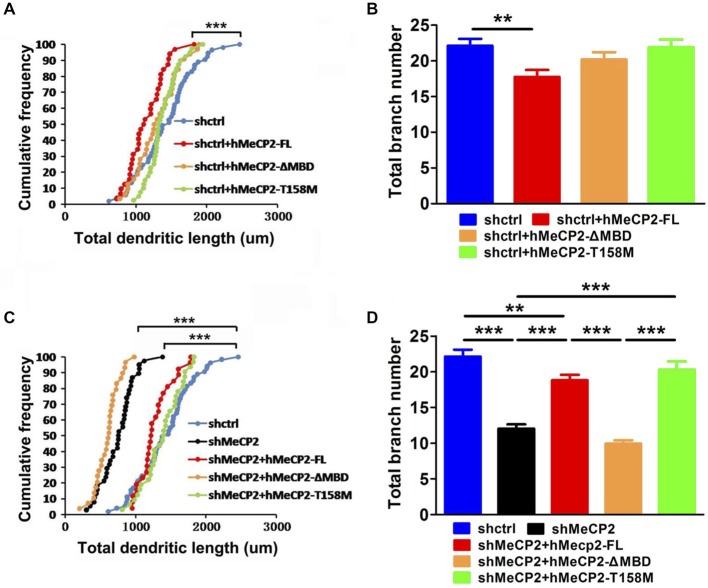
**Full length MeCP2 is essential for the maintenance of normal dendritic development in hippocampal primary neurons. (A,B)** Graphs showing total dendritic length **(A)** and total branch number **(B)** of cells at 12 DIV after co-infection of various MeCP2 expressing virus (as indicated) in shctrl-expressing cells. **(C,D)** Graphs showing total dendritic length **(C)** and total branch number **(D)** of cells at 12 DIV after co-infection of various MeCP2 expressing virus (as indicated) in shMeCP2-expressing cells. (**p < 0.01 and ***p < 0.001, one way ANOVA with a *post hoc* Tukey multiple comparison, number of neurons in **(A)** and **(B)**: *n* = 51 (shctrl), 32 (shctrl + hMeCP2-FL), 33 (shctrl + hMeCP2 − ΔMBD) and 40 (shctrl + hMeCP2-T158M); number of neurons in **(C)** and **(D)**: *n* = 51 (shctrl), 38 (shMeCP2), 26 (shMeCP2 + hMeCP2-FL), 29 (shMeCP2 + hMeCP2 − ΔMBD), 32 (shMeCP2 + hMeCP2-T158M) from at least 3 batches).

We next determined if an intact MBD in MeCP2 is essential for the maintenance of normal dendritic development in cultured hippocampal neurons. Co-expression of hMeCP2-FL but not hMeCP2-ΔMBD or hMeCP2-T158M with shctrl, moderately decreased the total dendritic length and branch number in control neurons (Figures 2A,B), indicating a loss of function of both hMeCP2-ΔMBD and hMeCP2-T158M. As expected, the total dendritic length and total branch number were significantly decreased in MeCP2-silenced neurons (***p < 0.001) (Figures 2C,D), indicating that MeCP2 is necessary for dendrite development of these neurons. However, only the expression of hMeCP2-FL and hMeCP2-T158M in MeCP2 silenced neurons were effective in rescuing the MeCP2-deficiency-associated dendritic outgrowth defects (Figures 2C,D). In contrast, hMeCP2-ΔMBD overexpression had no effects on MeCP2 silenced neurons (Figures 2C,D). This indicates hMeCP2-T158M may still have residual functions as compared to hMeCP2-ΔMBD.

### Intact MBD Domain in MeCP2 is Essential for Synapse Formation

**Figure 3:**
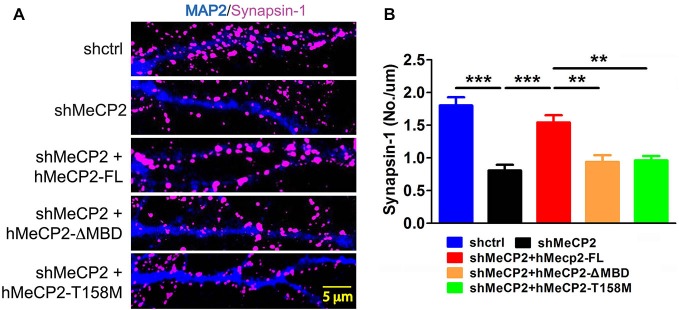
**Full length MeCP2 is required for synapse formation. (A)** Representative immunostaining images of subcellular distribution of synapsin-1 (pink) in cells infected with shctrl or shMeCP2, co-expression of shMeCP2 with hMeCP2-FL, hMeCP2-ΔMBD or hMeCP2-T158M. **(B)** Quantification of synapsin-1 puncta density per μm dendrite of neurons infected with the constructs as indicated on the graph. (**p < 0.01 and ***p < 0.001, one-way ANOVA with a *post hoc* Tukey multiple comparison, number of neurons in **(A)** and **(B)**: *n* = 15 (shctrl), 18 (hMeCP2), 13 (shMeCP2 + hMeCP2-FL), 16 (shMeCP2 + hMeCP2-ΔMBD), 15 (shMeCP2 + hMeCP2-T158M) from at least 3 batches).

We next determined if defects in dendritic development lead to impaired synapse formation in cultured hippocampal neurons. These neurons were immunostained for synapsin-1 (synaptic vesicle protein) to gauge the number of synapses. Quantification of the colocalized puncta densities was performed along MAP2-labeled dendrites. There was a significant decrease in the density of the synapsin-1 puncta in MeCP2-silenced neurons compared to control neurons (Figures 3A,B). Expression of hMeCP2-FL in these MeCP2-silenced neurons significantly blocked the decrease in density of synapsin-1 puncta (Figure 3B). In contrast, expression of hMeCP2-ΔMBD or hMeCP2-T158M in MeCP2-silenced neurons did not result in any significant changes of the number of synaptic puncta (Figure 3B). These data indicate that an intact MBD domain in MeCP2 is essential for synapse formation in primary hippocampal neurons.

### MeCP2 Modulates Spontaneous Calcium Oscillations in Hippocampal Neurons

**Figure 4:**
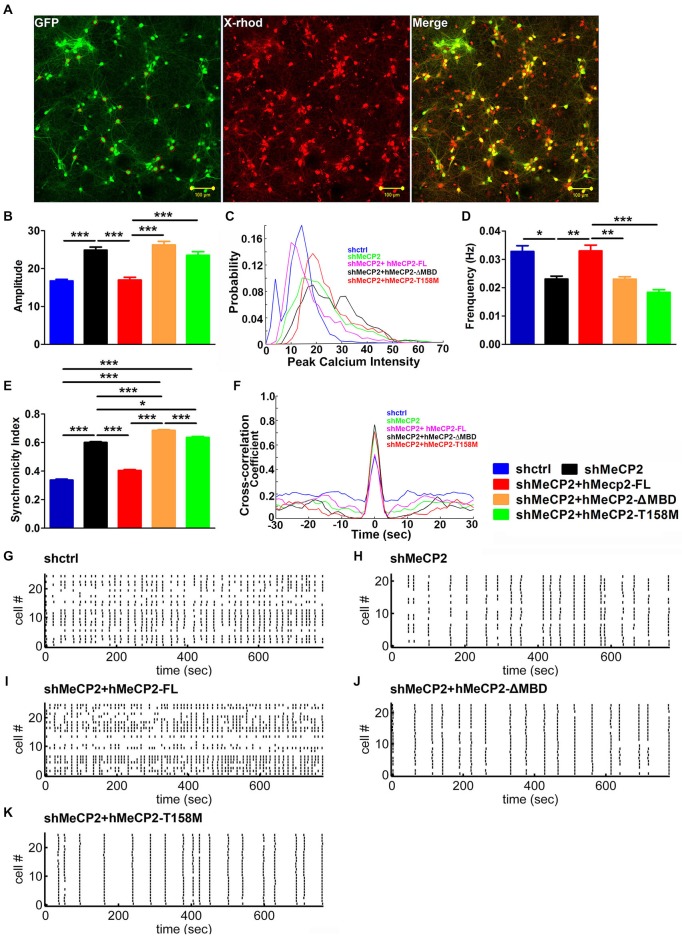
**MeCP2 regulates spontaneous calcium oscillations in primary hippocampal neurons. (A)** Representative images of neurons pre-infected with different kind of virus (green) and stained with X-Rhod-1 (red) (scale bar = 100 μm). **(B–F)** Graph shows the amplitude of calcium oscillations **(B)**, the distribution probability of amplitude **(C)**, the frequency **(D)** and the synchronicity index **(E)** as well as the cross-correlation coefficient **(F)** of calcium oscillations in neurons infected with the indicated constructs. **(G–K)** Representative Spike Raster Plot of the different groups of neurons as indicated, showing calcium spikes from >20 neurons/plot over a time period of 800 s as indicated on the *X*-axis. (*p < 0.05, **p < 0.01 and ***p < 0.001, one-way ANOVA with a *post hoc* Tukey multiple comparison, *n* = 120 neurons from at least 3 batches).

To investigate if MeCP2 is also involved in neurotransmission and synaptic functions, we monitored Ca^2+^ oscillations in primary hippocampal neurons using a fluorescent calcium dye (X-Rhod-1) to examine the activity of many neurons simultaneously (Figure 4A). Neuronal activity is associated with spontaneous and synchronous rises in intracellular calcium concentration (calcium spikes) in these hippocampal neurons (Leinekugel et al., 1997; Ma et al., 2015). MeCP2-silenced neurons showed increase amplitude but decrease frequency of the spontaneous Ca^2+^ oscillations compared to control neurons (Figures 4B,C,D,G,H). Cross-correlation analysis demonstrated a higher synchronicity index in MeCP2 silenced neurons compared to control neurons (Figures 4E–H), indicating stronger synchronization of activity upon MeCP2 silencing.

We then examined the changes in spontaneous Ca^2+^ oscillations after expression of hMeCP2-FL, hMeCP2-ΔMBD or hMeCP2-T158M in MeCP2-silenced neurons. Expression of hMeCP2-FL in MeCP2-silenced neurons rescued MeCP2 deficiency-mediated defects in the amplitude (Figure 4B), the frequency (Figure 4D) and the synchronicity index (Figures 4E,F) of spontaneous Ca^2+^ oscillations when compared to MeCP2-silenced neurons alone (Figures 4B–K). However, expression of neither hMeCP2-ΔMBD nor hMeCP2-T158M in MeCP2-silenced neurons could rescue the abnormal spontaneous Ca^2+^ oscillation pattern caused by MeCP2 silencing (Figures 4B–K).

### Cell Autonomous Effects of MeCP2 in Dendritic Development of Adult Newborn Neurons Requires the MBD Domain

**Figure 5:**
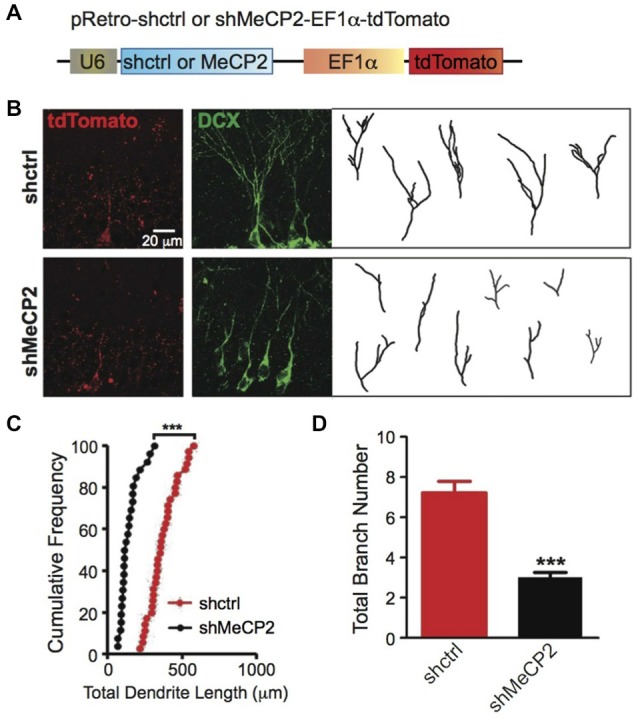
**MeCP2-regulated dendritic morphology of newborn DG cells in the adult brain. (A)** Schematic diagram showing retroviral construct expressing shctrl or shMeCP2. **(B)** Representative images and tracings from confocal three-dimensional (3D) reconstruction of dendrites of shctrl and shMeCP2-expressing newborn neurons in the DG of adult brain (Scale bar = 20 μm). **(C)** Quantification of total dendritic length of shctrl or shMeCP2-expressing neurons. Each symbol represents a single neuron. **(D)** Quantification of total branch number of shctrl or shMeCP2-expressing neurons. (***p < 0.001, Student’s *t*-test, *n* = 35 neurons for shctrl and 26 neurons for shMeCP2 groups, from 5 mice per experimental group).

To examine cell autonomous effects of MeCP2 in adult newborn neurons, we stereotaxically injected retrovirus with shRNA against a scrambled control sequence or specifically against MeCP2 (Figure 5A) into the dentate gyrus of adult mice. We found significant morphological defects in dendritic arborization in MeCP2-silenced newborn neurons demonstrated by shorter total dendritic length (Figures 5B,C) and fewer branch numbers (Figures 5B,D) compared to control newborn neurons.

**Figure 6:**
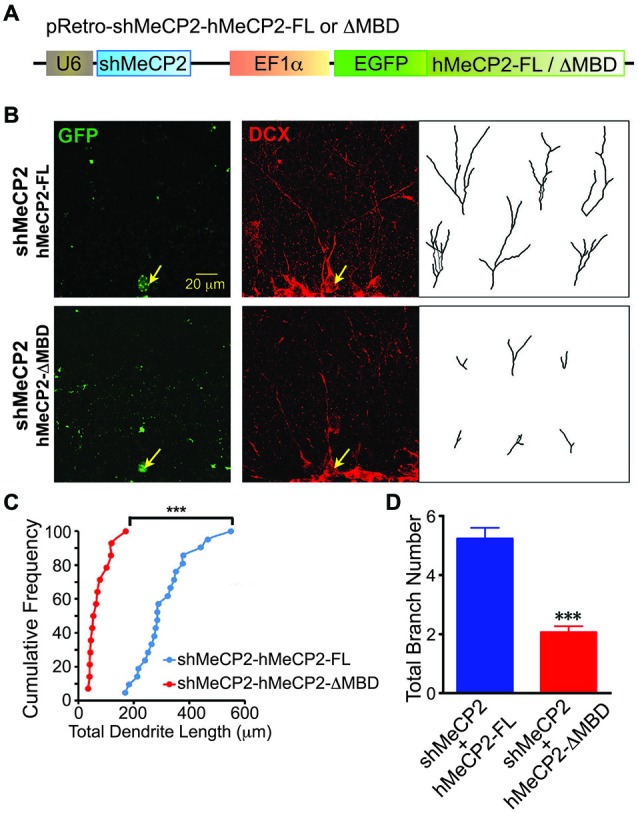
**Overexpression of hMeCP2-FL but not hMeCP2-ΔMBD rescued the defective dendritic morphologies of MeCP2-silenced newborn DG neurons in the adult brain. (A)** Schematic diagram showing retroviral construct expressing shMeCP2 together with EGFP-tagged hMeCP2-FL or ΔMBD. **(B)** Representative images and tracings from confocal three-dimensional (3D) reconstruction of dendrites of adult newborn neurons expressing either hMeCP2-FL-shMeCP2 or hMeCP2-ΔMBD-shMeCP2 (Scale bar = 20 μm). **(C)** Quantification of total dendritic length of hMeCP2-FL-shMeCP2 or hMeCP2-ΔMBD-shMeCP2-expressing adult newborn neurons in DG. Each symbol represents a single neuron. **(D)** Quantification of total branch number of hMeCP2-FL-shMeCP2 or hMeCP2-ΔMBD-shMeCP2-expressing adult newborn neurons in DG. (***p < 0.001 Student’s *t*-test, *n* = 21 neurons for hMeCP2-FL-shMeCP2 and 14 neurons for or hMeCP2-ΔMBD-shMeCP2 groups, from 5 mice per experimental group).

To confirm the role of MBD of MeCP2 in regulating the dendritic development of newborn neurons in adult brain, we coexpressed hMeCP2-FL or hMeCP2-ΔMBD with shMeCP2 (Figure 6A) in the same newborn granule neurons *in vivo*. hMeCP2-FL but not hMeCP2-ΔMBD expression in MeCP2-silenced neurons rescued MeCP2 knockdown-mediated dendritic length (Figures 6B,C) and branching (Figures 6B,D) phenotypes. These results confirm the essential role of MBD in dendritic outgrowth of newborn neurons in adult dentate gyrus *in vivo*.

## Discussion

Disturbances in brain development, neuronal morphology and connectivity were observed in the RTT mouse models (Chen et al., 2001; Belichenko et al., 2008; Lyst and Bird, 2015). Here we provide morphological and functional evidence for the MBD-dependent role of MeCP2 in neuronal development in cultured hippocampal neurons and in newborn granule neurons in adult hippocampus. Our data showed that knockdown of MeCP2 affects both dendritic length and branch complexity in hippocampal neurons both *in vitro* (Figure 2) and *in vivo* (Figure 5). These observations suggest that MeCP2 is crucial in promoting dendritic growth during early neuronal development. Since morphological alterations in brain architecture are subtle in MeCP2 mutants, there is increasing focus on the functional aspects of synaptic signaling impairments in RTT models. Previous electrophysiological studies on adult mutant MeCP2 mice and MeCP2 knockdown cells revealed an enhanced excitatory neurotransmission (Moretti et al., 2006; Ma et al., 2015) and reduced inhibitory synaptic responses in GABAergic neurons (Chao et al., 2010). Here our results demonstrate a reduction of synapse density in cultured hippocampal neurons upon knocking down MeCP2. Expression of hMeCP2-FL but not the other two MBD mutants in MeCP2-silenced neurons enhanced synapse density, indicating synaptic dysfunction is a critical contributor to RTT phenotypes. Furthermore, it was reported that an imbalance between excitatory and inhibitory neurotransmission is responsible for several neuropsychiatric phenotypes exhibiting altered learning and memory (Cui et al., 2008) and impaired social behavior (Tabuchi et al., 2007).

Spontaneous Ca^2+^ oscillations occur as a result of periodical increase and decrease of the free Ca^2+^ (Garaschuk et al., 2000; Berridge et al., 2003; Okubo et al., 2011), and have been shown to occur in many different neuronal types and at different stages of maturation (Owens et al., 2000; Marchetto et al., 2010; Linde et al., 2011) and are thought to play critical roles in neuronal development and plasticity (Clapham, 1995; Jaskova et al., 2012). A previous study also showed that spontaneous Ca^2+^ oscillations encode information in their frequency to regulate neurotransmitter expression, channel maturation and neurite extension in spinal neurons (Gu and Spitzer, 1995). The pattern of spontaneous Ca^2+^ oscillations was significantly altered with increased amplitude and decreased frequency in our cultured MeCP2-silenced hippocampal neurons. MeCP2 knockdown also results in synchronized Ca^2+^ oscillations among neurons, which suggest that the underlying electrical activity, and this functional coupling is correlated to the morphological appearance. However, only hMeCP2-FL but not the other 2 MBD mutants rescued these deficiencies caused by MeCP2 knockdown.

Patients with mutations in the MBD exhibited more severe clinical features than the other mutations that occurred outside this area (Fabio et al., 2014). Mutation of T158, located at the C terminus of the MBD in MeCP2, is one of the most common mutations observed in RTT (Goffin et al., 2011). Approximately 10% of RTT cases carry the mutation of T158 to methionine or, in rare cases, alanine (Bienvenu and Chelly, 2006). Expression of hMeCP2-ΔMBD upon MeCP2 knockdown did not rescue any defects exhibited in MeCP2 silenced neurons. Intriguingly, expression of hMeCP2-T158M upon MeCP2 knockdown only rescued dendritic arborization (branching), but not the other MeCP2-dysfunction mediated defects that we examined. MeCP2 is named for its ability in binding to specific methylated CpG dinucleotides and MBD is solely responsible for this binding (Yusufzai and Wolffe, 2000; Ghosh et al., 2010). Furthermore, MBD is highly conserved and appears to be the central hub for MeCP2 tertiary structure, forming contacts with the NTD, ID and the TRD. Previous experiments have established the critical role for T158 in the binding of MeCP2 to methylated DNA *in vitro* (Hansen et al., 2011). Consistently, the T158 mutation was found to have reduced affinity of MeCP2 for methylated DNA *in vivo* and MeCP2^T158A/y^ mice also presented RTT-like symptoms, including altered anxiety, breathing abnormalities, and impaired learning and memory (Goffin et al., 2011).

Taken together, we found that knocking down MeCP2 in hippocampal neurons resulted in morphological and physiological deficiencies including alterations in dendrite length and branching as well as the number of synaptic puncta and the spontaneous calcium oscillations. These data indicate an essential role of MeCP2 in synaptic functions. The MeCP2 with T158 mutation only rescued dendritic branching defects while MBD domain truncated deletion of MeCP2 is a complete loss-of function for these MeCP2-dysfunction mediated defects examined. The study of specific mutations or alterations in *Mecp2* gene is important for understanding functional implications of these changes in RTT. In addition, our *in vivo* data also showed cell autonomous effects in dendrites length and branching caused by MeCP2 deficiency that can only be rescued by the full length MeCP2 but not MeCP2-ΔMBD. Thus, indicating that the MBD domain in MeCP2 is critical for normal dendrite development *in vitro* and *in vivo*.

## Author Contributions

NZ performed most *in vitro* and *in vivo* experiments, analyzed data and wrote the manuscript; DM designed all experiments, performed some *in vitro* and *in vivo* experiments and calcium imaging experiments; WYL designed and made all MeCP2 WT and mutant constructs; JH and AVD analyzed data for calcium imaging; TC provided inputs to the project; ELG initiated and directed the entire study, designed experiments, analyzed data and wrote the manuscript.

## Conflict of Interest Statement

The authors declare that the research was conducted in the absence of any commercial or financial relationships that could be construed as a potential conflict of interest.
